# Antibiotic prescribing and antimicrobial stewardship in long-term care facilities: Past interventions and implementation challenges

**DOI:** 10.14745/ccdr.v48i1112a04

**Published:** 2022-11-03

**Authors:** Niyati Vyas, Tyler Good, Jorida Cila, Mark Morrissey, Denise Gravel Tropper

**Affiliations:** 1 Antimicrobial Resistance Task Force, Infectious Disease Prevention and Control Branch, Public Health Agency of Canada, Ottawa, ON; 2 Office of Behavioural Science, Corporate Data and Surveillance Branch, Public Health Agency of Canada, Ottawa, ON

**Keywords:** antimicrobial stewardship, antibiotic stewardship, antibiotic prescribing, long-term care, long-term care facilities, nursing homes, antimicrobial resistance

## Abstract

**Background:**

The threat of antimicrobial resistance (AMR) is rising, leading to increased illness, death and healthcare costs. In long-term care facilities (LTCFs), high rates of infection coupled with high antibiotic use create a selective pressure for antimicrobial-resistant organisms that pose a risk to residents and staff as well as surrounding hospitals and communities. Antimicrobial stewardship (AMS) is paramount in the fight against AMR, but its adoption in LTCFs has been limited.

**Methods:**

This article summarizes factors influencing antibiotic prescribing decisions in LTCFs and the effectiveness of past AMS interventions that have been put in place in an attempt to support those decisions. The emphasis of this literature review is the Canadian LTCF landscape; however, due to the limited literature in this area, the scope was broadened to include international studies.

**Results:**

Prescribing decisions are influenced by the context of the individual patient, their caregivers, the clinical environment, the healthcare system and surrounding culture. Antimicrobial stewardship interventions were found to be successful in LTCFs, though there was considerable heterogeneity in the literature.

**Conclusion:**

This article highlights the need for more well-designed studies that explore innovative and multifaceted solutions to AMS in LTCFs.

## Introduction

Antimicrobial resistance (AMR) is a global health emergency with rising human and financial costs (1). The threat is especially pertinent in long-term care facilities (LTCFs), which provide a range of healthcare options to older adults unable to live independently in the community, ranging from resident and long-term care to post-acute rehabilitation care (2). Older adults living in LTCFs are often clinically frail and at high risk of infection and subsequent antibiotic use (3,4). The leading indications for antibiotic use in LTCFs were urinary tract infections (UTIs), lower respiratory tract infections (LRTIs) and skin and soft tissue infections (SSTIs) (5). Of these, suspected UTIs provided the greatest challenge to antimicrobial stewardship (AMS), with up 70.5% of antibiotic prescriptions being considered clinically unnecessary, compared with 55.7% of prescriptions for LRTI and 22.0% for SSTI (5). While antibiotics are indispensable tools for combatting serious infections, inappropriate use, in terms of initiation, duration or dose, increases the possibility of selecting antimicrobial-resistant organisms (AROs) (3,6). Long-term care facilities can become reservoirs for AROs, threatening the well-being of LTCF residents and staff as well as the surrounding hospital and community (7-9).

## Methods

Antimicrobial stewardship programs have been implemented in some LTCFs, often leading to reduced prevalence of AROs and improved resident outcomes (10). However, there was a paucity of reviews from a Canadian perspective examining these AMS programs. This article describes factors influencing antibiotic prescribing decisions and the effectiveness of AMS interventions that have attempted to support those decisions. The emphasis of this literature review is on the Canadian LTCF landscape; however, due to the limited number of studies performed in Canada, we included international studies as well. The Embase, Medline and Global Health databases were searched to identify relevant articles published prior to April 2022 (see Appendix for a complete list of search terms). This search resulted in 26 primary research articles examining factors affecting antibiotic prescribing (seven Canadian) (6,11-16) and 22 articles assessing the success of AMS interventions in LTCFs (four Canadian) (17-20). The overwhelming majority of these studies occurred in LTCFs or nursing homes, though one of the studies examining factors affecting antibiotic prescribing queried staff in assisted living facilities (21) and another included a sample of five nursing homes and two residential care facilities (22). Of the AMS intervention studies we assessed, two were implemented in skilled nursing facilities (23,24), while another studied assisted living facilities (25).

## Factors influencing antibiotic prescribing in long-term care facilities

**Figure 1:**
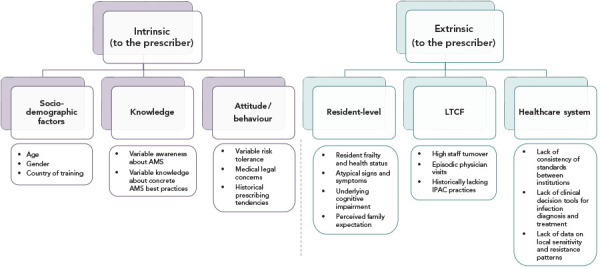
Antibiotic prescribing decisions are affected by factors intrinsic and extrinsic to the prescriber Abbreviations: AMS, antimicrobial stewardship; IPAC, infection prevention and control; LTCF, long-term care facility

Prescribing decisions are influenced by the context of the individual patient, their caregivers, the clinical environment, the healthcare system and the society that surrounds the prescriber. **Figure 1** summarizes the evidence for barriers to AMS in LTCFs that operate at each level.

### Prescriber factors

Antibiotic prescribing habits are highly variable among prescribers in LTCFs, and this variability is not accounted for by differences in resident characteristics (6) suggesting that individual prescribers have a role in driving antibiotic use and overuse. Past prescribing behaviour is a strong predictor of future prescribing (6), and being older, male and having completed medical school outside of Canada are associated with higher levels of antibiotic prescribing (6). Furthermore, tendency towards risk aversion (i.e. risk of delayed treatment and associated consequences) also influence antibiotic prescribing decisions (9,14,22,26).

Research also suggests that knowledge about AMR is variable in physicians and nurses and that knowledge gaps are associated with inappropriate prescribing (16,27,28). The search did not identify articles examining AMR knowledge in non-regulated caregivers, who provide much of the primary care in LTCFs.

### Resident population factors

Residents of LTCFs are an increasingly frail population with complex care needs (29,30). Medical complaints from LTCF residents often present with non-specific or atypical symptoms that create diagnostic uncertainty, posing a challenge to confident antibiotic prescribing (13,21,22,28,31). Furthermore, a high proportion of residents have underlying cognitive impairment that limits their ability to communicate the specific symptoms and disease course that would inform diagnosis (13,21,22,28,31). Caregivers, who are important advocates for residents, may be perceived as having expectations that can influence antibiotic prescribing decisions (16,32-34).

### Long-term care facilities environmental factors

Staffing patterns also contribute to antibiotic prescribing practices in LTCFs. Physicians visit LTCFs episodically, causing reliance on asynchronous communication strategies (i.e. fax, email, calls), which may lead to care team members not having the information they need to prescribe judiciously (9,27,28,31,32,35,36). High nursing and personal support worker turnover are also a major barrier to AMS in the LTCFs (16), perpetuating knowledge gaps among staff from lack of stability. Moreover, effective infection prevention and control practices, which are recognized to limit the spread of AMR, have historically been lacking in LTCFs due to limited resources and training opportunities (8,16,37-39). Prescribers may also perceive pressure due to medical legal concerns associated with adverse patient outcomes following the decision not to initiate an antibiotic prescription (38).

### Healthcare systems factors and surveillance

At the healthcare system level, lack of access to resident-relevant information and consistency of standards between different healthcare institutions are key factors impeding informed decision-making in antibiotic prescribing (16,36,38). While many hospitals have robust antibiogram programs, LTCFs lack data on local sensitivity or resistance patterns. In fact, most specimens collected from LTCFs are processed in private laboratories in Canada and antimicrobial susceptibility data from those sites are not always made available to prescribers, leaving them without local resistance determinants to inform prescribing (*personal communication, RP Rennie*). There is also a lack of specific guidelines or clinical decision tools regarding infection diagnosis and treatment for LTCF residents (14,22,28,35,38); these gaps impede informed antibiotic decision-making and ultimately increase the risk of selecting for AROs (22,28). Lastly, there are limited antibiotic surveillance data from Canadian LTCFs and an absence of data on appropriate use, which represent a missing foundation for AMS programs in the sector.

## Effectiveness of antimicrobial stewardship interventions in long-term care facilities

A variety of AMS intervention approaches in LTCF have been reported, with most articles testing multiple methods. Of the 22 articles reviewed, twelve used educational strategies and clinical practice guidelines (17,18,20,22,23,35,40-45). Others used a range of strategies, including audit and feedback (18,19,44,46-48), clinical care pathways (25,41,44), modified urine culture reporting (49), use of an infectious disease team (43,47,50,51) and interventions tailored to local needs (18,23,42,43). There was no single AMS intervention best practice; instead, articles have shown generally positive, but heterogeneous, results for many approaches. The AMS interventions most commonly targeted physicians (18,19,23,42,46,47,50,51) or both physicians and nurses (17,22,24,25,35,44,48,52). It was less common for AMS trials to focus solely on nurses (40,41,45,53), pharmacists (22,52), caregivers (25,43,44) or residents (44). Antimicrobial stewardship intervention approaches were reported only rarely in Canada; four of the 22 articles were implemented in Canadian LTCFs (17-20).

In the following sections, the results from these 22 articles are summarized and organized by outcome measure.

### Antibiotic prescribing

Available evidence suggests that AMS interventions have generally been effective in reducing antibiotic prescribing, with a recent meta-analysis finding interventions associated with a 14% overall reduction in antimicrobial use (AMU) (10). Primary research points to the positive effects of AMS interventions in reducing antibiotic prescriptions, especially for the treatment of UTIs (20,45,53). It should be noted that outcomes assessing the appropriateness of antibiotic prescriptions are a more precise measure of stewardship than AMU; however, collecting these data is more labour-intensive and fewer articles examined this outcome measure (18,22,2324,41,46,52). Among the studies that did measure the appropriateness of antibiotic prescriptions, the evidence was mixed; with some showing statistically significant improvements (18,41,46) and others not (22-24,52). Another important study outcome was the duration of therapy, where deprescribing interventions (i.e. the planned process of reducing or stopping medications that are no longer needed or may be causing harm) showed promise (54). Two articles showed reductions in the duration of antibiotic therapy following an AMS intervention (19,48), but more research is needed in this area.

### Balancing measures

A recent systematic review found AMS interventions did not increase hospital admissions or deaths, indicating that these programs did not lead to under-treatment of infections (55). There was still limited evidence in this area and a need for further study. Future AMS articles should continue to monitor the safety of interventions by tracking mortality and morbidity outcomes as well as appropriateness measures.

## Special focus on urinary tract infection

Antibiotic prescribing for suspected UTIs is a primary focus of AMS in LTCF. At the core of this challenge is the diagnosis of asymptomatic bacteriuria, which has a remarkably high incidence among LTCF residents (3,56). The judicious use of diagnostic tools for UTIs plays an important role in supporting UTI treatment decisions. The practice of routine dipstick analysis regardless of UTI symptoms increased the frequency of antibiotic use despite the known lack of utility of these tests among LTCF residents (22,38). Dipstick analysis is generally not recommended for LTCF residents (57); however, the rate of de-adoption is unknown. Only one article examined this outcome and it did not show a decrease in the use of dipstick analysis following an AMS intervention that included the education of staff about new clinical practice guidelines through AMS program champions (40).

An upstream focus on the judicious use of urine cultures may be helpful in reducing unnecessary antibiotic prescriptions for UTIs given the high rates of asymptomatic bacteriuria in the LTCF population. Three articles have taken this approach, all showing a successful reduction in urine cultures, as well as, importantly, AMU (13,20,48). The timing of microbiology test results was also relevant, as delayed results increased the use of antibiotics, especially when coupled with increased risk aversion in the prescriber (16,22,31,32,38). Lastly, providing prescribers with local annual antibiograms may also be effective in reducing the rate of urine cultures and urinary antibiotics (58).

## Discussion

Antimicrobial resistance is a public health threat with considerable health and economic burden (3) and a serious health-related issue for LTCF residents (7,59). Available evidence points to multiple factors influencing antibiotic overprescribing in LTCFs operating at various levels. These range from 1) individual differences in health care workers’ knowledge of AMS to 2) variability in risk tolerances in nurses and doctors to 3) lack of consistent clinical guidelines and to 4) established practices (e.g. dipstick analysis). A significant issue in the Canadian context is the lack of institutional surveillance on AMU and local resistance patterns, which is foundational to successful AMS programs. Published articles showed that the adoption of AMS interventions in LTCFs can be effective, albeit with significant variability in effect sizes. Meaningful, sustainable implementation of AMS programs in LTCFs will require multifaceted solutions that address barriers faced by different decision-makers in the system.

The most frequently used interventions in AMS programs were educational components and clinical practice guidelines; however, there was no consensus on one specific strategy for an effective stewardship program, as no single intervention generated sufficient, sustainable improvement in antibiotic prescribing (60,61). Multifaceted AMS interventions at different levels could help reduce unnecessary or inappropriate AMU, ensure the optimal selection of antimicrobial therapies (i.e. dosage and duration) and help impede selective pressure for AROs (9,10). Implementation of a multifaceted AMS intervention would require dedicated resources in LTCFs (9). The practice of behavioural science has at its core a focus on changing behaviour—a foundational pillar of AMS. In other sectors, including acute care hospitals and community, behavioural science trials have been successful in delivering impactful, low-cost components to AMS programs (62,63). Heavier-handed solutions, like antibiotic restriction policies, may also play a role in enforcing stewardship, though their implementation must be carefully considered (64).

In the Canadian context, barriers to AMS partly reflect a historical and ongoing under-emphasis of vulnerable older adults, which manifests as poorly funded institutions with substandard working conditions, and a struggle to attract and retain a stable and qualified workforce—a situation only made more precarious during the coronavirus disease 2019 pandemic. A more thorough examination of social and cultural drivers of AMS in Canada has been conducted by other researchers (65).

The literature documents many barriers to AMS in LTCFs, with a particularly strong focus on factors that affect prescribers. This is crucial given the integral role these clinicians play in AMS; however, there is room for further study of the perspectives of non-prescribing healthcare providers on AMS, who provide most of the primary care in LTCFs (e.g. registered nurses, registered practical nurses, and personal support workers) and who are often the first to identify infections within the residents of LTCFs. A study of the diverse stakeholders in LTCFs may reveal novel opportunities for a broader set of individuals to participate in stewardship. Additionally, the relative importance and interconnectedness of barriers are unclear and further study is needed to parse the potential benefits of AMS interventions focused on each part of the system. A multifaceted problem warrants a multifaceted approach. Learning from the hospital sector (66), systems dynamics modelling may provide an important role on this front, as outcomes in non-linear systems like LTCFs are difficult to predict with conventional methods. Most of the articles assessing AMS effectiveness also rely on small sample sizes, limiting generalizability, which is particularly relevant given a heterogeneous LTCF landscape. Finally, we note that there is limited national-level surveillance data on AMU and AMR in Canadian LTCFs, which is necessary to inform future AMS efforts.

### Conclusion

This article identified a wide range of barriers to judicious antibiotic prescribing in LTCFs and summarized evidence that indicates that AMS programs can be effective in this environment. While this article focused on LTCFs, its findings may also be relevant to assisted living facilities as the resident populations in these settings are similar. Future work should consider perspectives from a diverse group of stakeholders to help uncover how a larger group of actors can be supported as allies in AMS in LTCFs. The development of further high-quality trials is also needed, especially in Canada, to help understand which interventions retain effectiveness over time and across the heterogeneous LTCF landscape. Finally, strengthening the national surveillance system for AMU and AMR in LTCFs in Canada will be foundational to measure the impact of AMS strategies in this challenging setting.

## Appendix

Table A1: Embase, 1974 to April 1, 2022

Table A2: Ovid MEDLINE(R) ALL, 1946 to April 1, 2022

Table A3: Global Health, 1973 to April 1, 2022

**Table A1:** Embase, 1974 to April 1, 2022

#	Search terms
1	*Antimicrobial Stewardship/
2	(antimicrobial stewardship or amr).ti,kw.
3	((stewardship* or "use" or misus* or abus* or overus* or therap* or prescrib*) and (antimicrobial* or antibiotic* or antibacterial* or antiviral* or antifungal* or anti microbial* or anti biotic* or anti bacterial* or anti viral* or anti fungal*)).ti,kw.
4	or/1-3 [AMR]
5	residential home/ or nursing home/ or assisted living facility/
6	(long term care facilit* or convalascence home or convalascence facilit* or nursing home? or group home? or assisted living or seniors home? or seniors residence? or old age home? or old age residence? or aged care home? or aged care residence? or residential facilit* or residential institution?).tw,kw.
7	(elder* or older adult? or old age? or seniors or geriatric).tw,kw.
8	or/5-7 [long term care facilities]
9	exp Clinical Audit/
10	(program* or intervention* or audit or feedback or prescriber education or pharmacist education).ti,kw. or (stewardship adj2 program*).ab. or (intervention* or audit or feedback or prescriber education).ab. /freq=2
11	or/9-10 [interventions]
12	exp health personnel attitude/
13	exp health care personnel/ or (doctor? or physician? or family practitioner? or clinician? or nurse? or nursing staff or personal support worker? or caregiver? or care giver? or health care professional? or Health Personnel or health care personnel or pharmacist?).tw,kw.
14	(perspective? or perception? or perceive? or believe? or belief? or view? or attitude? or opinion?).tw,kw.
15	and/13-14
16	or/12,15 [attitude of health personell]
17	4 and 8 and (11 or 16)
18	limit 17 to (english or french)

**Table A2:** Ovid MEDLINE(R) ALL, 1946 to April 1, 2022

#	Search terms
1	*Antimicrobial Stewardship/
2	(antimicrobial stewardship or amr).ti,kw,kf.
3	((stewardship* or "use" or misus* or abus* or overus* or therap* or prescrib*) and (antimicrobial* or antibiotic* or antibacterial* or antiviral* or antifungal* or anti microbial* or anti biotic* or anti bacterial* or anti viral* or anti fungal*)).ti,kw,kf.
4	or/1-3 [AMR]
5	exp Residential Facilities/
6	(long term care facilit* or convalascence home or convalascence facilit* or nursing home? or group home? or assisted living or seniors home? or seniors residence? or old age home? or old age residence? or aged care home? or aged care residence? or residential facilit* or residential institution?).tw,kw,kf.
7	(elder* or older adult? or old age? or seniors or geriatric).tw,kw,kf.
8	or/5-7 [long term care facilities]
9	exp Clinical Audit/
10	(program* or intervention* or audit or feedback or prescriber education or pharmacist education).ti,kw,kf. or (stewardship adj2 program*).ab. or (intervention* or audit or feedback or prescriber education).ab. /freq=2
11	or/9-10 [interventions]
12	exp "attitude of health personnel"/
13	exp Health Personnel/ or (doctor? or physician? or family practitioner? or clinician? or nurse? or nursing staff or personal support worker? or caregiver? or care giver? or health care professional? or pharmacist?).tw,kw,kf.
14	(perspective? or perception? or perceive? or believe? or belief? or view? or attitude? or opinion?).tw,kw,kf.
15	and/13-14
16	or/12,15 [attitude of health personell]
17	4 and 8 and (11 or 16)
18	limit 17 to (english or french)

**Table A3:** Global Health, 1973 to April 1, 2022

#	Search terms
1	(antimicrobial stewardship or amr).ti,hw.
2	((stewardship* or "use" or misus* or abus* or overus* or therap* or prescrib*) and (antimicrobial* or antibiotic* or antibacterial* or antiviral* or antifungal* or anti microbial* or anti biotic* or anti bacterial* or anti viral* or anti fungal*)).ti,hw.
3	or/1-2 [AMR]
4	residential institutions/ or nursing home/ or long term care/
5	(long term care facilit* or convalascence home or convalascence facilit* or nursing home? or group home? or assisted living or seniors home? or seniors residence? or old age home? or old age residence? or aged care home? or aged care residence? or residential facilit* or residential institution?).tw,hw.
6	(elder* or older adult? or old age? or seniors or geriatric).tw,hw.
7	or/4-6 [long term care facilities]
8	(program* or intervention* or audit or feedback or prescriber education or pharmacist education).ti,hw. or (stewardship adj2 program*).ab. or (intervention* or audit or feedback or prescriber education).ab. /freq=2
9	exp health care workers/ or (doctor? or physician? or family practitioner? or clinician? or nurse? or nursing staff or personal support worker? or caregiver? or care giver? or health care professional? or Health Personnel or health care personnel or pharmacist?).tw,hw.
10	(perspective? or perception? or perceive? or believe? or belief? or view? or attitude? or opinion?).tw,hw.
11	and/9-10
12	3 and 7 and (8 or 11)
13	limit 12 to (english or french)
